# Effect of Chitosan, Cymbopogon citratus, and Ultraviolet-C Disinfection on the Surface Accuracy and Dimensional Stability of Polyvinyl Ether Silicone (PVES) Impression Material: An In Vitro Comparative Study

**DOI:** 10.7759/cureus.111823

**Published:** 2026-06-30

**Authors:** Sravanthi Ennala, Vimal Bharathi, Praveen M, Meghana V, Maram Kavya, Saguna Kaul

**Affiliations:** 1 Prosthodontics, Army College of Dental Sciences, Secunderabad, IND; 2 Prosthodontics, Panineeya Institute of Dental Sciences and Research Centre, Hyderabad, IND; 3 Prosthodontics, Mamata Institute of Dental Sciences, Hyderabad, IND

**Keywords:** dimensional stability, elastomeric impression material, impression disinfection, surface detail reproduction, uv-c radiation, vinyl polyether silicone

## Abstract

Background

Dental impressions often become contaminated during clinical use and must be disinfected before laboratory handling to prevent cross-infection. Disinfection methods, however, can influence key properties such as surface detail reproduction (SDR) and linear dimensional stability (LDS). Polyvinyl ether silicone (PVES), a hybrid elastomer, offers strong dimensional accuracy and hydrophilic behavior.

Aim

This study investigated the effects of 0.5% chitosan, 30% *Cymbopogon citratus* (lemongrass), and ultraviolet-C (UV-C) radiation on the SDR and LDS of PVES impressions.

Materials and methods

Seventy-two PVES specimens were prepared under American National Standards Institute (ANSI)/American Dental Association (ADA) No. 19 and International Organization for Standardization (ISO) 4823 standards, then randomly divided into four groups: control (Group I), 0.5% chitosan (Group II), 30% *C. citratus *(Group III), and UVC radiation (Group IV), each with 18. Groups II and III were immersed in disinfectant solutions for 10 minutes, while Group IV was exposed to UV-C light (254 nm) for the same period. SDR was examined under a stereomicroscope (×10), and LDS was measured with a digital vernier caliper. Readings were taken at 15 minutes (T1) and 12 hours (T2). Statistical analysis employed chi-square, one-way ANOVA, and repeated-measures ANOVA, with p<0.05 considered significant.

Results

No statistically significant differences were observed among the groups for SDR at T1 (p=0.428) or T2 (p=0.320). The UV-C group demonstrated the highest frequency of ideal SDR scores at both evaluation periods. Similarly, LDS values were comparable among all groups, with no significant differences at T1 (p=0.558) or T2 (p=0.976). All disinfected groups exhibited dimensional measurements within clinically acceptable limits.

Conclusions

Disinfection of PVES impression material using 0.5% chitosan, 30% *C. citratus*, and UV-C radiation did not adversely affect surface detail reproduction or dimensional stability. Among the tested methods, UV-C radiation demonstrated the most favorable surface detail reproduction, while *C. citratus* showed dimensional stability comparable to the control group. All three disinfection protocols may be considered suitable for routine clinical use.

## Introduction

Accurate dental impressions are essential for the fabrication of precise indirect restorations and prostheses. The success of an impression depends largely on the properties of the impression material used. The popularity of elastomeric impression materials stems from their dimensional consistency, elasticity, and capacity to replicate fine surface detail. Polyvinyl ether silicone (PVES) is a recently introduced hybrid elastomeric material that combines the dimensional accuracy of polyvinyl siloxane with the hydrophilic properties of polyether [1]. Owing to its improved wettability and flow characteristics, PVES is considered suitable for recording fine details, especially in moist clinical conditions [2].

Dental impressions are frequently contaminated with saliva, blood, dental plaque, and microorganisms during clinical procedures, making them potential vehicles for cross-contamination among dentists, dental assistants, laboratory personnel, and patients [3,4]. Studies have demonstrated the presence of pathogenic microorganisms, including *Streptococcus* species, *Staphylococcus aureus*, *Candida albicans*, hepatitis viruses, and other blood-borne pathogens on untreated impressions, emphasizing the need for effective disinfection before transfer to the dental laboratory [3-5]. Therefore, disinfection of impressions before laboratory transfer is considered mandatory to minimize the risk of disease transmission. Commonly used disinfectants include sodium hypochlorite, glutaraldehyde, iodophors, phenols, ethanol, and isopropanol, which can be applied using spray or immersion techniques [4]. However, prolonged exposure to disinfectants may alter the physical properties of impression materials, particularly dimensional stability and surface detail reproduction [5]. Hence, an ideal disinfectant should effectively eliminate microorganisms without compromising impression accuracy.

Natural and alternative disinfectants have recently gained attention because of their antimicrobial efficacy and reduced toxicity. Chitosan is a naturally derived biopolymer obtained from crustacean shells and exhibits broad-spectrum antimicrobial activity due to its interaction with microbial cell walls [6]. *Cymbopogon citratus* (lemongrass) is a plant-derived disinfectant that exhibits broad-spectrum antimicrobial activity. Studies confirm its effectiveness against oral bacteria like *Streptococcus mutans* and *S. aureus* and fungi such as *C. albicans*, suggesting its suitability as a natural disinfectant [7]. Ultraviolet-C (UV-C) radiation is another effective disinfection method that inactivates microorganisms by damaging microbial DNA at wavelengths between 200 and 280 nm, with 254 nm commonly used in dental UV chambers [8].

Considering the increasing use of alternative disinfectants, the present in vitro study aimed to evaluate and compare the effects of 0.5% chitosan, 30% *C. citratus*, and UV-C radiation on the surface detail reproduction (SDR) and linear dimensional stability (LDS) of PVES impression material.

## Materials and methods

This in vitro study was conducted in the Prosthodontics Department at Panineeya Mahavidyalaya Institute of Dental Sciences, Hyderabad, India. The study was approved by the Institutional Ethics Committee of the Panineeya Mahavidyalaya Institute of Dental Sciences and Research Centre (RC/IEC/PROS/DN/456-21).

Sample size calculation

The sample size was estimated using G*Power software version 3.1.9.7 (Heinrich Heine University, Düsseldorf, Germany) for a one-way analysis of variance (ANOVA) with fixed effects. An effect size (f) of 0.51, representing a large effect based on Cohen's classification, was assumed to detect clinically meaningful differences among the four study groups. Considering an alpha error probability of 0.05 and a study power of 95% (1−β = 0.95), the analysis yielded a minimum sample size of 72 specimens (numerator df = 3, denominator df = 68), corresponding to an actual power of 95.57%. Therefore, a total of 72 specimens were included and equally allocated among the four groups, with 18 specimens per group.

Preparation of specimens

Specimens were fabricated in accordance with American National Standards Institute (ANSI)/American Dental Association (ADA) Specification No. 19 and International Organization for Standardization (ISO) 4823 standards for elastomeric impression materials [9]. The stainless steel master die consisted of three horizontal lines measuring 25 μm, 50 μm, and 75 μm in width, intersected by two perpendicular reference lines.

The PVES impression material was mixed and dispensed according to the manufacturer's instructions using an automixing dispensing system. The material was injected into the mold assembly, covered with a glass plate to ensure uniform thickness, and allowed to polymerize completely under standardized laboratory conditions.

A total of 72 specimens were fabricated, coded numerically, and randomly allocated into four groups (n = 18/group) using a computer-generated randomization sequence created with the randomization function in Microsoft Excel 2019 (Microsoft Corp., Redmond, Washington, United States). The allocation sequence was generated by an investigator not involved in specimen evaluation, and group assignments were concealed from the blinded examiner responsible for assessing surface detail reproduction and dimensional stability.

Group I served as the control group and received no disinfection treatment. Group II specimens were disinfected using a 0.5% aqueous chitosan hydrochloride solution (Mahtani Chitosan Pvt. Ltd., Veraval, Gujarat, India), while Group III specimens were treated with a 30% commercially available aqueous *C. citratus* extract (Fame Pvt. Ltd., Meerut, Uttar PRadesh, India). Both disinfectants were used as supplied by the manufacturers without further modification. Group IV specimens were subjected to UV-C radiation disinfection. This allocation ensured equal distribution of specimens among the experimental groups for subsequent evaluation of surface detail reproduction and dimensional stability.

Disinfection protocol

For Groups II and III, specimens were completely immersed in freshly prepared disinfectant solutions for 10 minutes at room temperature. Following immersion, the specimens were rinsed with distilled water for 30 seconds and gently air-dried [10]. For Group IV, specimens were placed in a UV-C chamber and exposed to UV radiation at a wavelength of 254 nm for 10 minutes. The specimens were positioned at a standardized distance of approximately 10 cm from the UV source to ensure uniform exposure [11,12]. The control group received no disinfection treatment.

Evaluation Time Intervals

All specimens were evaluated at two predetermined time intervals: (i) T1: 15 minutes after impression making, (ii) T2: 12 hours after impression making. During the interval period, specimens were stored under standardized laboratory conditions at room temperature.

Assessment of LDS

LDS was assessed by measuring the distance between the two vertical reference lines of the master die using a digital vernier caliper with an accuracy of 0.01 mm.

Three consecutive measurements were obtained for each specimen by a single calibrated examiner, and the mean value was recorded for analysis. Dimensional change was determined by comparing the measured values with the corresponding dimensions of the master die.

Assessment of SDR

SDR was evaluated according to ANSI/ADA Specification No. 19 using a stereomicroscope at 10× magnification. The continuity and sharpness of the 50 μm line were assessed by a blinded examiner using a four-point ordinal scoring system adapted from the method described by Khatri et al. [10]. To minimize observer bias, all specimens were examined by a single blinded evaluator under standardized magnification and illumination conditions. Specimens exhibiting a sharp and continuous line were assigned Score 1; those showing slight loss of sharpness, Score 2; those demonstrating marked deterioration or discontinuity, Score 3; and those failing to reproduce the line, Score 4.

Statistical analysis

Data were entered into Microsoft Excel and analyzed using IBM SPSS Statistics for Windows, version 22.0 (Released 2013; IBM Corp., Armonk, New York, United States). Normality was assessed using the Shapiro-Wilk test, and descriptive statistics were expressed as mean, standard deviation, frequency, and percentage. SDR scores were analyzed using the Chi-square test to compare the distribution of categorical outcomes among the study groups. As most observations were concentrated within Scores 1 and 2, while Scores 3 and 4 exhibited zero frequencies across all groups, these categories were retained in the analysis to maintain consistency with ANSI/ADA Specification No. 19. LDS was evaluated using one-way ANOVA, followed by post hoc comparisons when appropriate. A p-value <0.05 was considered statistically significant.

## Results

Intergroup comparison of SDR scores was performed using the Chi-square test. At both T1 and T2, most specimens in all groups demonstrated Score 1, indicating optimal surface detail reproduction, with no specimens exhibiting Scores 3 or 4. No statistically significant differences were observed among the groups at T1 (p=0.428) or T2 (p=0.320), suggesting that 0.5% chitosan, 30% *C. citratus*, and UV-C disinfection did not significantly affect the SDR of the PVES impression material (Table 1).

**Table 1:** Intergroup comparison of surface detail reproduction at T1 and T2 Chi-square test; p<0.05 considered statistically significant.

Timeline	Group	Score 1	Score 2	Score 3	Score 4	P value	χ² value
T1	Group I (Control)	18	0	0	0	0.428	1.868
	Group II (0.5% Chitosan)	13	5	0	0		
	Group III (30% Cymbopogon citratus)	14	4	0	0		
	Group IV (UV-C)	17	1	0	0		
T2	Group I (Control)	18	0	0	0	0.320	2.442
	Group II (0.5% Chitosan)	9	9	0	0		
	Group III (30% Cymbopogon citratus)	13	5	0	0		
	Group IV (UV-C)	17	1	0	0		

Intergroup comparison of LDS at T1 was performed using one-way ANOVA. Although minor variations in mean dimensional measurements were observed among the groups, the differences were not statistically significant (p=0.558). The control group demonstrated the highest mean value, while the UV-C group showed the lowest mean value. Overall, 0.5% chitosan, 30% *C. citratus*, and UV-C disinfection did not significantly affect the dimensional stability of the PVES impression material at T1 (Table 2).

**Table 2:** Intergroup comparison of linear dimensional stability at T1 One-way ANOVA; p<0.05 considered statistically significant.

Groups	Mean ± SD	Standard Error	P value	F value
Group I (Control)	22.000 ± 0.00	0.000	0.558	0.811
Group II (0.5% Chitosan)	21.984 ± 0.18	0.043		
Group III (30% *Cymbopogon citratus)*	21.994 ± 0.07	0.017		
Group IV (UV-C)	21.944 ± 0.16	0.038		

Intergroup comparison of LDS at T2 was performed using one-way ANOVA. The mean dimensional measurements were comparable across all groups, with only minimal variations observed. No statistically significant differences were found among the groups (p=0.976), indicating that 0.5% chitosan, 30% *C. citratus*, and UV-C disinfection maintained the dimensional stability of the PVES impression material after 12 hours (Table 3).

**Table 3:** Intergroup comparison of linear dimensional stability at T2 One-way ANOVA; p<0.05 considered statistically significant.

Groups	Mean ± SD	Standard Error	P value	F value
Group I (Control)	22.000 ± 0.00	0.000	0.976	0.151
Group II (0.5% Chitosan)	22.012 ± 0.22	0.051		
Group III (30% *Cymbopogon citratus*)	22.008 ± 0.13	0.031		
Group IV (UV-C)	22.025 ± 0.21	0.052		

## Discussion

During clinical use, dental impressions often come into contact with saliva, blood, and oral microbes, creating a risk of cross-contamination among patients, staff, and laboratory personnel [13,14]. Therefore, effective disinfection of impressions is an indispensable component of infection control protocols. However, the selected disinfectant should not adversely affect the physical properties of impression materials, particularly SDR and LDS, as these properties directly influence the accuracy and fit of indirect restorations and prostheses [15].

PVES is a relatively recent hybrid elastomeric impression material developed to combine the hydrophilic nature of polyether with the dimensional accuracy and elastic recovery of polyvinyl siloxane [16]. Previous studies have demonstrated that PVES exhibits superior wettability, excellent detail reproduction, and favorable dimensional stability when compared with conventional elastomeric impression materials. Nassar et al. reported that PVES maintained excellent dimensional stability even after prolonged storage periods, supporting its clinical reliability for indirect restorative procedures [17]. Similarly, Ahuja et al. demonstrated that contemporary PVES materials retain dimensional accuracy following exposure to commonly used disinfection protocols [18].

The present study evaluated the effects of 0.5% chitosan, 30% *C. citratus*, and UV-C radiation on the SDR and LDS of PVES impression material at 15 minutes (T1) and 12 hours (T2) following impression making. The 15-minute interval was selected to represent the earliest clinically feasible time for pouring impressions after disinfection, allowing adequate elastic recovery of the impression material. The 12-hour interval was chosen to simulate delayed pouring conditions commonly encountered in clinical practice, such as transportation to dental laboratories or overnight storage, and to assess the dimensional stability of PVES over an extended period [19]. The findings revealed that all tested disinfection methods maintained clinically acceptable SDR and LDS, with no statistically significant differences when compared with the control group.

With respect to SDR, the UV-C group demonstrated the highest proportion of specimens exhibiting ideal surface detail reproduction at both evaluation periods. Although the differences were not statistically significant, this observation may be attributed to the non-contact mechanism of UV-C disinfection. Unlike immersion-based disinfectants, UV-C radiation does not expose the impression material to chemical agents or moisture that could potentially alter the surface characteristics of the polymer matrix. UV-C exerts its antimicrobial effect through the formation of pyrimidine dimers within microbial DNA, thereby inhibiting microbial replication without inducing direct physicochemical changes in the impression material. The absence of liquid contact may contribute to the preservation of surface characteristics and dimensional integrity of elastomeric impression materials. Similar observations have been reported in studies evaluating UV disinfection of dental impression materials, where UV exposure produced minimal alterations in physical properties while maintaining impression accuracy. Furthermore, Joshi et al. demonstrated that UV disinfection resulted in clinically insignificant dimensional changes and preserved the dimensional stability of elastomeric impression materials within ISO-recommended limits [19]. These findings support the use of UV-C radiation as a promising alternative disinfection modality for PVES impression materials.

Chitosan demonstrated slightly lower SDR scores when compared with the UV-C and *C. citratus* in this study. Chitosan, a polysaccharide produced by deacetylating chitin, is recognized for its wide antimicrobial activity, biocompatibility, and minimal toxicity [20]. Chitosan’s antimicrobial effect arises from its positively charged amino groups binding to negatively charged microbial membranes, disrupting permeability and causing cell death. The minor reduction in SDR seen in this study may be related to the hydrophilic nature of both the aqueous chitosan solution and the PVES material itself. Limited water sorption or superficial interaction with the impression surface may have influenced the reproduction of extremely fine details. Nevertheless, all specimens remained within clinically acceptable limits, indicating that chitosan did not adversely affect the clinical performance of the impression material.

*C. citratus* demonstrated favorable performance in both SDR and LDS evaluations in this study. Lemongrass possesses well-documented antimicrobial activity against oral microorganisms owing to the presence of bioactive compounds such as citral, geraniol, limonene, and other terpenoid constituents [21]. Almeida et al. reported significant antibacterial and antifungal activity of *C. citratus* against *Staphylococcus* species, *S. mutans*, and *Candida *species, supporting its potential application as a natural disinfecting agent [22]. The favorable dimensional stability observed in the present study suggests that the herbal extract did not induce significant physicochemical alterations within the polymeric network of PVES. This finding supports the growing interest in plant-derived disinfectants as environmentally friendly alternatives to conventional chemical agents.

Assessment of LDS revealed only minimal dimensional changes among all experimental groups at both evaluation periods. Although slight variations were observed following disinfection, none of the differences reached statistical significance. These findings indicate that the structural integrity of the PVES polymer network remained largely unaffected by immersion in chitosan or *C. citratus* solutions or by exposure to UV-C radiation. The solvent composition of the disinfectants may influence the dimensional behavior of elastomeric materials; however, the absence of significant dimensional alterations in the present study suggests that the vehicles used did not adversely affect the stability of PVES under the tested conditions. Similar observations have been reported by Melilli et al. and Pal et al., who concluded that immersion disinfection of elastomeric impression materials resulted in negligible dimensional changes that were clinically insignificant [23,24].

Among the disinfected groups, the *C. citratus* group demonstrated dimensional measurements closest to those of the control group at both time intervals. This may be attributed to the relatively mild interaction of herbal extracts with the impression material surface. In contrast, the slight dimensional variations observed in the chitosan group may be associated with limited water uptake from the aqueous disinfectant solution, particularly considering the hydrophilic characteristics of PVES. Similar mechanisms have been proposed by Carvalhal et al. and Rios et al., who suggested that water sorption may contribute to minor dimensional alterations in elastomeric impression materials following immersion disinfection [25,26]. Nevertheless, the dimensional changes observed in the present study remained well within clinically acceptable limits.

The results of the present investigation are also supported by recent evidence. A systematic review and meta-analysis by Hassan et al. concluded that chemical disinfection procedures generally produce only minimal dimensional alterations in PVES impression materials and that such changes are unlikely to compromise clinical accuracy [27]. Furthermore, three-dimensional analyses conducted by Soganci et al. demonstrated that elastomeric impression materials maintain acceptable dimensional accuracy following immersion disinfection, further supporting the findings of the present study [28].

From a clinical perspective, maintaining both SDR and LDS following disinfection is essential because inaccuracies in impressions can adversely affect the marginal adaptation, retention, and longevity of indirect restorations. The present study demonstrated that all three tested disinfection methods preserved the critical physical properties of PVES impression material. Therefore, these disinfection protocols may be incorporated into routine infection-control procedures without compromising impression accuracy.

This study was conducted under in vitro conditions, which may not completely replicate the complex oral environment and clinical handling procedures. Only one hybrid elastomeric impression material (PVES) and a single disinfection cycle were evaluated. Although the effects of disinfection on SDR and LDS were investigated, the antimicrobial efficacy of the tested disinfectants was not assessed. Additionally, examiner calibration and reliability statistics for surface detail reproduction scoring were not determined, which may have introduced a degree of subjective bias despite blinded evaluation. Future investigations should evaluate the antimicrobial efficacy of these disinfectants against clinically relevant oral microorganisms, assess different concentrations and exposure times, and investigate additional properties such as tear strength, wettability, surface roughness, and three-dimensional accuracy using digital scanning technologies.

## Conclusions

Within the limitations of this in vitro study, disinfection of PVES impression material with 0.5% chitosan, 30% *C. citratus*, and UV-C radiation did not significantly affect surface detail reproduction or linear dimensional stability at 15 minutes and 12 hours after impression making. These findings suggest that the evaluated eco-friendly disinfectants and UV-C radiation may serve as promising alternatives for impression disinfection while preserving the dimensional accuracy of PVES. Further studies evaluating their antimicrobial efficacy and clinical performance are recommended.

## References

[1] Papazoglou E, Wee AG, Carr AB, Urban I, Margaritis V (2020). Accuracy of complete-arch implant impression made with occlusal registration material. J Prosthet Dent.

[2] Siadat H, Saeidi Z, Alikhasi M, Zeighami S (2018). Comparative evaluation of the effect of impression materials and trays on the accuracy of angulated implants impressions. J Clin Exp Dent.

[3] Marya CM, Shukla P, Dahiya V, Jnaneswar A (2011). Current status of disinfection of dental impressions in Indian dental colleges: a cause of concern. J Infect Dev Ctries.

[4] (1996). Infection control recommendations for the dental office and the dental laboratory. ADA Council on Scientific Affairs and ADA Council on Dental Practice. J Am Dent Assoc.

[5] Qiu Y, Xu J, Xu Y, Shi Z, Wang Y, Zhang L, Fu B (2023). Disinfection efficacy of sodium hypochlorite and glutaraldehyde and their effects on the dimensional stability and surface properties of dental impressions: a systematic review. PeerJ.

[6] Elsaka SE (2012). Antibacterial activity and adhesive properties of a chitosan-containing dental adhesive. Quintessence Int.

[7] Kiełtyka-Dadasiewicz A, Esteban J, Jabłońska-Trypuć A (2024). Antiviral, antibacterial, antifungal, and anticancer activity of plant materials derived from Cymbopogon citratus (DC.) Stapf species. Pharmaceuticals (Basel).

[8] Wezgowiec J, Wieczynska A, Wieckiewicz M (2022). Evaluation of antimicrobial efficacy of UVC radiation, gaseous ozone, and liquid chemicals used for disinfection of silicone dental impression materials. Materials (Basel).

[9] (1977). Revised American Dental Association specification no. 19 for non‑aqueous, elastomeric dental impression materials. J Am Dent Assoc.

[10] Khatri M, Mantri SS, Deogade SC (2020). Effect of chemical disinfection on surface detail reproduction and dimensional stability of a new vinyl polyether silicone elastomeric impression material. Contemp Clin Dent.

[11] Malpartida-Carrillo V, Tinedo-López PL, Salas-Quispe JE, Fry-Oropeza MA, Amaya-Pajares S, Özcan M (2024). Effect of ultraviolet C light disinfection on the dimensional stability of dental impression materials: a scoping review of the literature. J Clin Exp Dent.

[12] Sabharwal N, Arora A, Upadhyaya V (2024). Impression disinfection and its effect on dimensional accuracy and surface detail in the times of COVID- 19: an in vitro study. Cureus.

[13] Szymańska J, Sitkowska J, Dutkiewicz J (2008). Microbial contamination of dental unit waterlines. Ann Agric Environ Med.

[14] Turkistani KA, Turkistani KA (2020). Dental risks and precautions during COVID-19 pandemic: a systematic review. J Int Soc Prev Community Dent.

[15] Mantena SR, Mohd I, Pradeep DK, Suresh SM, Ramaraju AV, Bheemalingeswara RD (2019). Disinfection of impression materials: a comprehensive review of disinfection methods. Int J Dent Mater.

[16] Shetty RM, Bhandari GR, Mehta D (2014). Vinyl polysiloxane ether: a breakthrough in elastomeric impression material. World J Dent.

[17] Nassar U, Oko A, Adeeb S, El-Rich M, Flores-Mir C (2013). An in vitro study on the dimensional stability of a vinyl polyether silicone impression material over a prolonged storage period. J Prosthet Dent.

[18] Ahuja BM, Pawashe KG, Sanyal PK (2024). Assessment of dimensional stability of novel VPES impression material at different time intervals with standard disinfectants. BMC Oral Health.

[19] Joshi S, Madhav VN, Saini RS (2024). Evaluation of the effect of chemical disinfection and ultraviolet disinfection on the dimensional stability of polyether impression material: an in-vitro study. BMC Oral Health.

[20] Chen CY, Chung YC (2012). Antibacterial effect of water-soluble chitosan on representative dental pathogens Streptococcus mutans and Lactobacilli brevis. J Appl Oral Sci.

[21] Kumar P (2021). Evaluation of Cymbopogon citratus as disinfectant and its effect on the dimensional stability of the resultant gypsum casts: an in vitro study. Univ J Dent Sci.

[22] Almeida RB, Akisue G, Cardoso LM, Junqueira JC, Jorge AOC (2013). Antimicrobial activity of the essential oil of Cymbopogon citratus (DC) Stapf on Staphylococcus spp., Streptococcus mutans and Candida spp. Rev Bras Plantas Med.

[23] Melilli D, Rallo A, Cassaro A, Pizzo G (2008). The effect of immersion disinfection procedures on dimensional stability of two elastomeric impression materials. J Oral Sci.

[24] Pal PK, Kamble SS, Chaurasia RR, Chaurasia VR, Tiwari S, Bansal D (2014). Evaluation of different disinfactants on dimensional accuracy and surface quality of type iv gypsum casts retrieved from elastomeric impression materials. J Int Oral Health.

[25] Carvalhal CI, Mello JA, Sobrinho LC, Correr AB, Sinhoreti MA (2011). Dimensional change of elastomeric materials after immersion in disinfectant solutions for different times. J Contemp Dent Pract.

[26] Rios MP, Morgano SM, Stein RS, Rose L (1996). Effects of chemical disinfectant solutions on the stability and accuracy of the dental impression complex. J Prosthet Dent.

[27] Hassan SA, Alshadidi AF, Aldosari LI, Heboyan A, Saini RS (2023). Effect of chemical disinfection on the dimensional stability of polyvinyl ether siloxane impression material: a systemic review and meta-analysis. BMC Oral Health.

[28] Soganci G, Cinar D, Caglar A, Yagiz A (2018). 3D evaluation of the effect of disinfectants on dimensional accuracy and stability of two elastomeric impression materials. Dent Mater J.

